# Vestibular Evoked Myogenic Potentials Reveal Impairments in Vestibular Nerve Pathways in Children with Attention Deficit Hyperactivity Disorder

**DOI:** 10.3390/audiolres16020059

**Published:** 2026-04-14

**Authors:** Dekun Gao, Jianyong Chen, Feng Li, Yao Chen

**Affiliations:** 1 Department of Otorhinolaryngology-Head and Neck Surgery, Zhongshan Hospital, Fudan University, Shanghai 200032, China; gao.dekun@zs-hospital.sh.cn; 2 Department of Otorhinolaryngology-Head and Neck Surgery, Xinhua Hospital, Shanghai Jiao Tong University School of Medicine, Shanghai 200092, China; chenjianyong@xinhuamed.com.cn; 3 MOE-Shanghai Key Laboratory for Children’s Environmental Health, Department of Developmental and Behavioural Pediatric & Child Primary Care, Xinhua Hospital, Shanghai Jiao Tong University School of Medicine, Shanghai 200092, China; 4 Department of Endocrinology, Genetics and Metabolism, Shanghai Children’s Medical Center, Shanghai Jiao Tong University School of Medicine, Shanghai 200127, China

**Keywords:** ADHD, children, cVEMP, oVEMP, vestibular nerve conduction pathway

## Abstract

Objective: This study aims to analyze the characteristics of vestibular evoked myogenic potentials (VEMPs) and evaluate specific vestibular nerve pathway impairments in children with Attention Deficit Hyperactivity Disorder (ADHD) compared to typically developing (TD) children. Methods: Forty-five children with ADHD and 34 TD children were recruited. All participants underwent comprehensive acoustic (ACS) and galvanic (GVS) VEMP examinations. To account for within-subject correlation, statistical analyses were performed at the subject level. Results: Children with ADHD exhibited prolonged P13 and N23 latencies in both ACS-cVEMP and GVS-cVEMP compared to TD children. For oVEMP, the N1 latency of ACS-oVEMP was significantly shorter in the ADHD group, and the interval was prolonged. Additionally, the absolute amplitude of ACS-cVEMP was significantly and markedly higher in children with ADHD. Conclusions: Children with ADHD exhibit functional abnormalities in both the saccule inferior vestibular nerve pathway (reflected by cVEMP) and the utricle superior vestibular nerve pathway (reflected by oVEMP). These impairments are primarily characterized by altered neural conduction latencies and hyperactive amplitude responses, providing valuable electrophysiological insights into vestibular dysfunction in ADHD.

## 1. Introduction

Attention Deficit Hyperactivity Disorder (ADHD) is a highly prevalent neurodevelopmental disorder in children. Beyond the core symptoms of inattention, hyperactivity, and impulsivity, children with ADHD frequently exhibit motor clumsiness, frequent falls, spatial orientation difficulties, and impaired postural control [1,2]. These motor and balance deficits are intrinsically linked to the vestibular system, a complex sensory network responsible for perceiving head position, stabilizing gaze, and maintaining dynamic body equilibrium [3]. Recent literature has increasingly highlighted the role of vestibular dysfunction in ADHD. Studies utilizing dynamic posturography and rotational testing have demonstrated notable developmental lags in the visual vestibular networks and impaired balance function in this population [4].

Professor Yufeng Wang’s team, utilizing vestibular system assessment methods, demonstrated a notable developmental lag in the visual ocular and visual vestibular systems in children with ADHD compared to a normal control group [5]. Professor Selina B M Shum identified impaired balance function in children with ADHD, affecting not only somatosensory and visual aspects but also the vestibular system [6]. However, the exact physiological integrity of the peripheral vestibular otolith organs and their specific neural pathways in children with ADHD remains largely underexplored. Vestibular evoked myogenic potentials (VEMPs) provide a non-invasive, objective neurophysiological assessment of these specific peripheral pathways. Conventionally, the cervical VEMP (cVEMP) reflects the functional integrity of the saccule and the inferior vestibular nerve pathway, whereas the ocular VEMP (oVEMP) evaluates the utricle and the superior vestibular nerve pathway [7]. While acoustic stimulation (ACS) assesses the entire pathway starting from the otolith organs [8], galvanic vestibular stimulation (GVS) bypasses the middle ear and mechanoreceptors to directly stimulate the vestibular nerve afferents [9,10].

To date, few studies have comprehensively evaluated both the superior and inferior vestibular nerve pathways in children with ADHD using a combination of ACS and GVS methodologies. Therefore, the present study aims to analyze the latency and amplitude characteristics of ACS/GVS-VEMPs in medication-naive children with ADHD. By conducting a rigorous subject-level statistical analysis, we aim to uncover potential specific impairments in these distinct vestibular nerve pathways, thereby providing novel electrophysiological insights into the neurodevelopmental state of children.

## 2. Methods

### 2.1. Study Participants and Ethical Approval

This study was conducted at the Department of Developmental and Behavioral Pediatrics and Child Healthcare from October 2022 to October 2023. The study protocol was reviewed and approved by the Institutional Review Board (IRB) of Xinhua Hospital, Shanghai Jiao Tong University School of Medicine (Approval Number: XHEC-D-2022-138). Written informed consent was obtained from the legal guardians of all participants prior to any testing.

We enrolled 45 children diagnosed with ADHD and 34 typically developing (TD) children as the control group. The diagnosis of ADHD was strictly based on the criteria outlined in the Diagnostic and Statistical Manual of Mental Disorders, Fifth Edition (DSM-5), supported by the semi-structured interview Kiddie Schedule for Affective Disorders and Schizophrenia Present and Lifetime Version (K-SADS-PL) [11]. Importantly, all children in the ADHD group were psychostimulant-naive; none had received methylphenidate or other psychotropic medications prior to vestibular testing.

The TD children met the following inclusion criteria: (1) no history of dizziness, vertigo, or headaches; (2) no history of neurological, psychiatric, or traumatic disorders; (3) no otologic diseases and normal hearing; and (4) absence of cognitive impairments (IQ screening > 70).

### 2.2. ACS-VEMP and GVS-VEMP Procedures

All electrophysiological tests were performed using the Neuropack MEB-9404C system (Nihon Kohden, Japan, Toyko). Prior to electrode placement, the local skin was thoroughly cleaned to ensure the impedance remained below 10 Ω with minimal bilateral difference.

For the ACS-cVEMP testing, acoustic stimulation consisted of 500 Hz short pure tones delivered at an intensity of 105 dB nHL. The rise and fall times were 1 ms with a 2 ms plateau. The high-pass and low-pass filters were set at 10 Hz and 500 Hz, respectively, with an analysis window of 0–60 ms. The stimulation rate was 5.1 Hz, and over 50 sweeps were averaged per trial. Two active recording electrodes were symmetrically placed on the upper-middle third of the bilateral sternocleidomastoid (SCM) muscles, with the reference electrode on the upper sternum and the ground electrode on the mid-forehead. Since cVEMP is an inhibitory response, participants were placed in a supine position and instructed to elevate their heads at a 30-degree angle and rotate their necks to the contralateral side to activate the SCM. Background muscle activity was monitored simultaneously, and muscle tension was maintained above 50 μV for children over 3 years old.

For the ACS-oVEMP testing, active electrodes were placed approximately 1 cm below the midpoint of the bilateral inferior orbital margins, with reference electrodes 2–3 cm below them. To record this crossed excitatory response from the inferior oblique muscle, participants remained in a supine position and were instructed to fixate on a blue target dot located 30–45 degrees upward-backward. Blinking was minimized to maintain stable inferior oblique muscle tension.

Galvanic vestibular stimulation (GVS) utilized a 5.0 mA, 1 ms direct current (DC) pulse delivered at a rate of 5 Hz. For the GVS-cVEMP, the recording amplifier was set with a bandpass filter of 20–2000 Hz and a 50 ms window, averaging 50 sweeps per reaction. Recording electrodes were placed similarly to ACS-cVEMP (active on mid-SCM, reference on suprasternal notch, ground on nasion). The DC stimulating cathode was placed on the mastoid, and the anode was positioned at the midline of the forehead hairline. The testing posture mirrored the head-elevated neck-rotation method used in ACS-cVEMP. To eliminate electrical and mechanical artifacts, waveforms recorded during muscle relaxation were subtracted from those recorded during muscle contraction.

For the GVS-oVEMP, the bandpass filter was adjusted to 1–1000 Hz. Recording electrodes were placed 0.5–1.0 cm below the lower eyelid (active) and 2.0 cm below that (reference). The DC stimulating anode was placed at the mid-hairline and the cathode on the mastoid. Participants maintained the same 30–45 degree upward gaze posture as in ACS-oVEMP. To exclude current interference, waveforms recorded during a downward gaze were subtracted from those recorded during an upward gaze.

### 2.3. Data Collection and Parameter Definitions

The elicitation of a cVEMP response was identified by a biphasic waveform comprising an initial downward positive peak appearing at approximately 13 ms (labeled as P13), followed by an upward negative peak at approximately 23 ms (labeled as N23). These potentials are strictly dependent on cervical muscle contraction and disappear upon muscle relaxation. Reproducibility was confirmed by continuous recording; a cVEMP was considered successfully elicited if typical, reproducible waveforms were obtained across three consecutive trials. Conversely, if no typical waveform could be identified across three trials at the same stimulation intensity, the cVEMP was considered absent. The latencies of P13 and N23 were defined as the duration (in ms) from the stimulus onset (0 ms) to the highest or lowest points of the respective peaks. The interpeak interval was calculated as the absolute difference between the P13 and N23 latencies.

Similarly, the elicitation of an oVEMP response was defined by the presence of an initial upward negative peak occurring at approximately 10 ms (labeled as N1), followed by a downward positive peak at approximately 15 ms (labeled as P1). A valid oVEMP response required the reproducibility of these typical N1 and P1 peaks across three consecutive trials at the specified stimulus intensity. The N1 and P1 latencies were measured from the stimulus onset to the respective peak points. The oVEMP interval was defined as the absolute time difference (in ms) between the P1 and N1 peaks. In both ACS and GVS modalities, peak-to-peak amplitudes and latencies were systematically extracted from the reproducible waveforms at maximum stimulation intensity for further statistical analysis.

### 2.4. Statistical Analysis

To address the critical assumption of independent observations in statistical testing, the bilateral ears of a single participant with a systemic neurodevelopmental condition cannot be treated as independent samples. Therefore, VEMP responses (latencies, intervals, and amplitudes) from the left and right ears were averaged for each participant. All subsequent statistical analyses were rigorously performed at the subject level (*N* = 45 for ADHD; *N* = 34 for TD).

Data analysis was performed using SPSS Statistics (IBM, Version 31.0.2.0, Armonk, NY, USA) and GraphPad Prism (LLC, Version 11.0.0, San Diego, CA, USA). Continuous variables are expressed as mean ± standard deviation (SD). The normality of the data distribution was assessed using the Shapiro Wilk test. Baseline demographics, such as sex distribution, were compared using Pearson’s Chi-square test. For VEMP parameters, independent samples *t*-tests (or Welch’s *t*-tests for data with unequal variances) were utilized to compare the differences between the ADHD and TD groups.

Given the numerous comparisons conducted across latency, interval, and amplitude parameters for four different VEMP paradigms, the Benjamini Hochberg False Discovery Rate (FDR) correction was applied to control for multiple testing. Adjusted *p*-values (pFDR) < 0.05 were considered statistically significant. Furthermore, to evaluate the clinical magnitude of the findings, effect sizes were calculated using Cohen’s d, with values of 0.2, 0.5, and 0.8 representing small, medium, and large effect sizes, respectively.

## 3. Results

### 3.1. Group Baseline Characteristics of the Participants

The study included a total of 79 participants: 45 children in the ADHD group and 34 in the TD control group. The ADHD group comprised 37 males and 8 females, with a mean age of 7.04 ± 1.31 years. The TD group consisted of 19 males and 15 females, with a mean age of 5.41 ± 2.09 years. Statistical comparisons revealed significant baseline differences between the two groups regarding sex distribution (Chi-square = 5.297, *p* = 0.021) and age (t = 3.997, *p* < 0.001). However, these discrepancies accurately reflect the well-established epidemiological and clinical profile of ADHD, which exhibits a significantly higher prevalence in males and is predominantly evaluated during early school age [12]. Therefore, this cohort remains a highly representative clinical sample for pediatric ADHD.

### 3.2. VEMP Elicitation Rates and Waveform Characteristics

**Figure 1:**
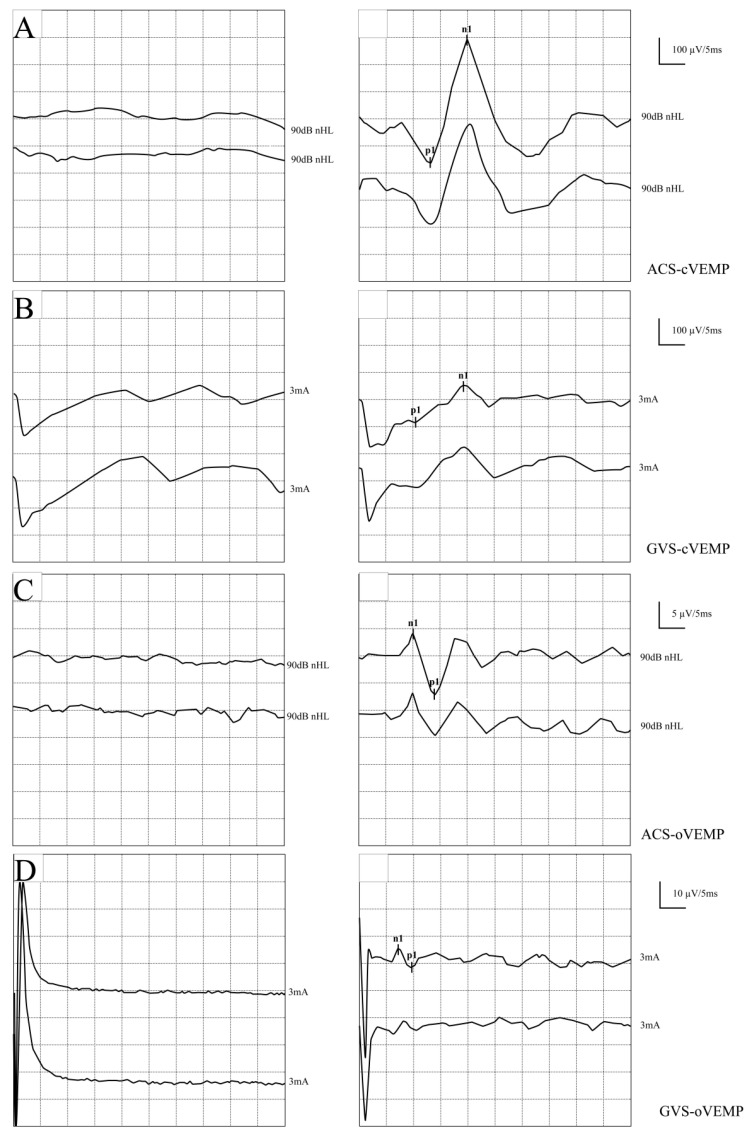
Representative waveform series of VEMPs elicited by different stimulation modalities in children with ADHD and typically developing (TD) children. Typical traces demonstrate the reproducible biphasic waveforms and distinct peak latency/amplitude characteristics for (**A**) ACS-cVEMP (p13–n23), (**B**) GVS-cVEMP (p13–n23), (**C**) ACS-oVEMP (n1–p1), and (**D**) GVS-oVEMP (n1–p1). The *x*-axis represents the time post-stimulation (ms), and the *y*-axis represents the rectified muscle activity amplitude (μV). VEMPs, vestibular evoked myogenic potentials; ACS, acoustic stimulation; GVS, galvanic vestibular stimulation.

Both acoustic (ACS) and galvanic (GVS) stimulation successfully elicited stable VEMP waveforms in the vast majority of participants. Typical ACS and GVS VEMP waveforms for both groups are illustrated in Figure 1. There were no statistically significant differences in the overall elicitation rates of ACS-cVEMP, GVS-cVEMP, ACS-oVEMP, and GVS-oVEMP between the ADHD and TD groups (all *p* > 0.05, as shown in Table 1 and Table 2 and Figure 1), indicating that the peripheral vestibular end-organs in ADHD children are highly responsive to both acoustic and electrical stimuli.

### 3.3. Impairments in the Saccule Inferior Vestibular Nerve Pathway (cVEMP)

To evaluate the saccule inferior vestibular nerve pathway, we compared the latencies, intervals, and amplitudes of cVEMPs between the two groups using a subject-level analysis (Table 3).

For ACS-cVEMP, children with ADHD exhibited significantly prolonged P13 latencies (15.67 ± 2.17 ms vs. 13.38 ± 1.29 ms, *p* < 0.001) and N23 latencies (23.33 ± 7.69 ms vs. 19.81 ± 1.58 ms, *p* = 0.018) compared to TD children. Similarly, GVS-cVEMP testing revealed a significantly prolonged P13 latency in the ADHD group (11.43 ± 1.61 ms vs. 10.61 ± 1.13 ms, *p* = 0.029). No significant differences were observed in the cVEMP intervals for either stimulation mode.

**Figure 2:**
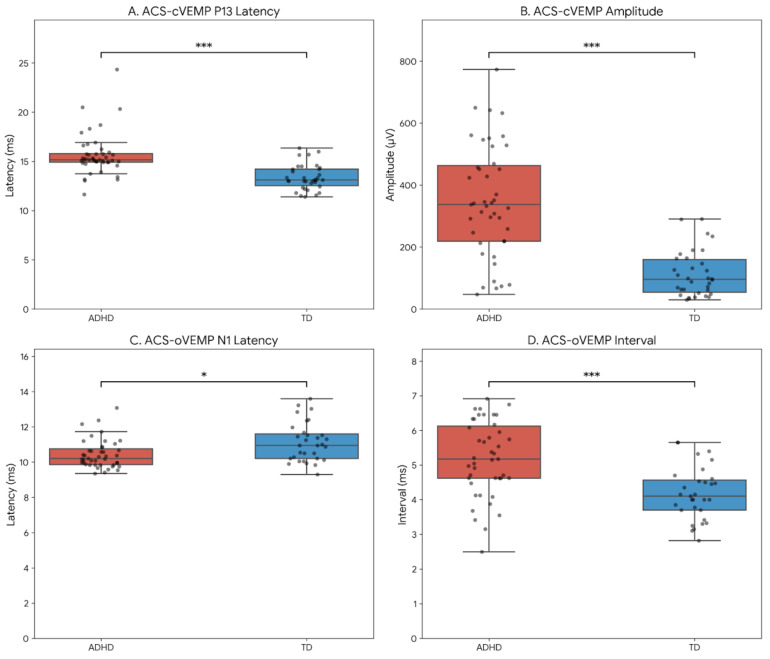
Statistical comparisons of critical VEMP parameters between children with ADHD and typically developing (TD) children. (**A**) Prolonged ACS-cVEMP P13 latency in the ADHD group. (**B**) Markedly increased ACS-cVEMP amplitude in the ADHD group. (**C**) Shortened ACS-oVEMP N1 latency in the ADHD group. (**D**) Prolonged ACS-oVEMP interval in the ADHD group. Box plots represent the median and interquartile ranges, while superimposed dots represent individual subject-level data points, illustrating data variability. Statistical significance was determined using independent *t*-tests with Benjamini Hochberg False Discovery Rate (FDR) correction (* *p* < 0.05; *** *p* < 0.001).

Strikingly, the absolute amplitude of ACS-cVEMP was drastically higher in the ADHD group (348.16 ± 181.27 μV) than in the TD group (112.74 ± 73.65 μV). This difference was highly significant (*p* < 0.001) and demonstrated an exceptionally large effect size (Cohen’s d = 1.63). These substantial differences in P13 latency and amplitude are visually highlighted in Figure 2A,B.

### 3.4. Alterations in the Utricle Superior Vestibular Nerve Pathway (oVEMP)

The utricle–superior vestibular nerve pathway, assessed via oVEMP, demonstrated a different pattern of alteration (Table 4). In ACS-oVEMP testing, the N1 latency was significantly shorter in the ADHD group compared to the TD group (10.45 ± 0.81 ms vs. 11.12 ± 1.11 ms, *p* = 0.018) (Figure 2C). Consequently, the ACS-oVEMP interval (P1-N1) was significantly prolonged in the ADHD group (5.18 ± 1.09 ms vs. 4.18 ± 0.76 ms, *p* < 0.001) (Figure 2D). For GVS-oVEMP, while latencies did not reach statistical significance after FDR correction, the GVS-oVEMP amplitude was significantly higher in children with ADHD (6.04 ± 2.69 μV) compared to TD children (4.69 ± 2.09 μV, *p* = 0.047), indicating a medium effect size (Cohen’s d = 0.55).

## 4. Discussion

ADHD is one of the most common disorders among school age children. Research has identified specific vestibular dysfunction in children with ADHD [6].

Postural and visual instability are clinical symptoms observed in individuals with ADHD, closely associated with the function of the vestibular system. The vestibular system serves various functions, primarily participating in spatial orientation and perception. Importantly, it plays a crucial role in maintaining postural and gait stability, visual stability, and stability in the autonomic nervous system.

The vestibular system consists of seven neural pathways (vestibulo-ocular, vestibulo-spinal, vestibular reticular formation, vestibulo-cerebellar, vestibulo-autonomic, vestibular cortex, and visual vestibular interaction) and three central control centers. These components furnish essential information for postural control across three dimensions of sensory integration [7]. Ayres’ Sensory Integration Theory proposes that the vestibular, proprioceptive, and tactile sensory systems play a significant role in sensory integration functions [13]. Individuals with ADHD who experience vestibular or proprioceptive processing issues may encounter difficulties in tracking moving targets or maintaining a stable field of vision. Consequently, this can have an impact on their reading and writing performance.

The vestibular pathway plays a role in the formation of abnormal brain networks in children with ADHD [14]. Various brain regions, including cortical and subcortical structures, participate in the perception and processing of vestibular information [15]. Impairments in these structures may further lead to the manifestation of symptoms such as hyperactivity and impulsivity [16]. Studies have identified altered activation states in ADHD patients within various brain regions, including the right frontal cortex, temporoparietal junction, insula, right supplementary motor area, precentral gyrus, right thalamus, and left caudate nucleus [17]. In recent years, advancements in vestibular testing techniques have shifted scholarly focus from the central nervous system to the peripheral vestibular system in ADHD research, leading to significant findings. Studies have uncovered various abnormalities in vestibular function tests in individuals with ADHD, such as increased vestibular ocular reflex (VOR) gain and impaired fixation ability. These issues may be linked to lesions in the central vestibular pathways, particularly those involving the cerebellum [18,19]. ADHD children undergoing vestibular rehabilitation have shown significant improvements in saccadic accuracy, smooth pursuit gain, VOR gain, Bruininks-Oseretsky Motor Proficiency Test scores, and choice reaction time [20]. Moreover, studies indicate an elevated risk of ADHD, sensory-motor disorders, and emotional, behavioral, and learning problems in late preterm infants (gestational age ≥ 34 weeks). VEMP abnormalities are proposed as potential contributors to these issues [21].

To our knowledge, this is one of the most comprehensive studies to simultaneously evaluate both the saccule–inferior and utricle–superior vestibular nerve pathways in children with ADHD using both ACS and GVS methodologies. By applying a rigorous subject-level statistical analysis to account for intra-subject correlation, our study reveals distinct vestibular impairments in children with ADHD, primarily characterized by delayed neural conduction in the saccule–inferior pathway and an overarching pattern of neuromuscular hypersensitivity.

**Impairments in the Saccule Inferior Vestibular Nerve Pathway**

Our results demonstrate that children with ADHD exhibit significantly prolonged P13 and N23 latencies in both ACS-cVEMP and GVS-cVEMP compared to typically developing (TD) children. Because cVEMP strictly assesses the saccule and the inferior vestibular nerve, the prolonged latencies under both acoustic and direct electrical stimulation suggest an intrinsic delay in neural conduction. This delay may stem from delayed myelination or immaturity within the reflex arc of the inferior vestibular pathway. Notably, these findings contrast with a previous study by Isaac et al., in which only 13 children with ADHD and 13 control children were included, with a cVEMP elicitation rate in the ADHD group of only 76.9% and no significant cVEMP latency differences in ADHD [14]. Lotfi Younes’ study included 34 children with ADHD and 30 control children. While the sample size was closer to ours, all ADHD children in that study were taking methylphenidate [18]. This discrepancy can likely be attributed to our larger sample size, strict subject-level statistical modeling, and the fact that our ADHD cohort was completely psychostimulant-naive (e.g., no methylphenidate), suggesting that the prolonged latencies observed here reflect the natural, unmedicated neurodevelopmental state of the disorder.

**The Amplitude Paradox and Neuromuscular Hypersensitivity**

A striking and somewhat paradoxical finding in our study was the massively increased amplitude of ACS-cVEMP in the ADHD group (with a large effect size, Cohen’s d = 1.63). Classically, peripheral vestibular dysfunction results in prolonged latencies accompanied by decreased amplitudes. The inverse tendency observed here—prolonged latencies coupled with sharply increased amplitudes—warrants deep consideration. We hypothesize that rather than peripheral vestibular hyperactivity, this reflects a state of central disinhibition or hyper-excitability typical in ADHD. The nervous system of children with ADHD is often in a hypersensitive state, leading to exaggerated motor reflex responses (action potentials) when exposed to intense stimuli [22].

However, this amplitude finding must be interpreted with caution due to a notable methodological limitation: the absence of background electromyographic (EMG) normalization. Because VEMP amplitude is strongly dependent on tonic muscle contraction levels, standard adult protocols recommend EMG normalization [23,24,25]. In our pediatric cohort of hyperactive children aged 4 to 10, maintaining a consistent, quantifiable level of cervical muscle contraction with continuous EMG monitoring was practically unfeasible and would have severely compromised test completion rates. The viewpoint proposed by Gedik-Soyuyuce et al. [26] emphasizes that the most crucial goal in assessing vestibular function in children is to maintain cooperation and complete a comprehensive test, rather than attempting to conduct as many different tests as possible. Consequently, the unusually high absolute cVEMP amplitudes in the ADHD group might partially reflect higher baseline muscle tension or involuntary spasms inherent to the hyperactivity of the disorder.

**Alterations in the Vestibular Nerve Pathway**

In contrast to the clear conduction delays in the inferior pathway, the utricle–superior vestibular nerve pathway (reflected by oVEMP) presented a more subtle and complex pattern. Our subject-level analysis revealed a significantly shortened N1 latency in ACS-oVEMP among ADHD children, with no significant differences in absolute amplitudes after FDR correction. This shortened latency might reflect a compensatory neuro-excitability within the superior vestibular nerve branch. The dichotomy between the inferior pathway (delayed) and the superior pathway (hyperactive/shortened) highlights the uneven neurodevelopmental trajectories of distinct neural circuits in ADHD.

**Baseline Demographics and Study Limitations**

We acknowledge that our study cohorts exhibited baseline differences in age and sex distribution, with the ADHD group being slightly older and predominantly male. However, this discrepancy accurately reflects the well-established epidemiological profile of ADHD, which has a significantly higher prevalence in males and is predominantly diagnosed during early school age. Therefore, our cohort remains a clinically representative sample. Beyond the aforementioned lack of EMG normalization, another limitation is the cross-sectional nature of this study. While our findings confirm baseline vestibular abnormalities, we did not assess post-treatment outcomes.

## Figures and Tables

**Table 1:** Comparison of cVEMP elicite rates between the ADHD group and the TD group.

Group	ACS-cVEMP				GVS-cVEMP			
	Elicite (Ears)	Not Elicite (Ears)	χ^2^	*p*	Elicite (Ears)	Not Elicite (Ears)	χ^2^	*p*
ADHD	86	4	0.24	0.625	90	0	/	/
TD	66	2			64	4		

**Table 2:** Comparison of oVEMP elicite rates between the ADHD group and the control group.

Group	ACS-oVEMP				GVS-oVEMP			
	Elicited (Ears)	Not Elicited (Ears)	χ^2^	*p*	Elicite (Ears)	Not Elicite (Ears)	χ^2^	*p*
ADHD	83	7	3.57	0.059	82	8	0.76	0.382
TD	56	12			59	9		

**Table 3:** Comparison of latencies and intervals of cVEMP and oVEMP between the ADHD and TD groups. Asterisks (*) indicate *p* < 0.05.

Parameter	VEMP	ADHD Group (*N* = 45)	TD Group (*N* = 34)	T-Value	*p*-Value (FDR)
P13/N1 (ms)	ACS-cVEMP	15.67 ± 2.17	13.38 ± 1.29	5.814	<0.001 *
	GVS-cVEMP	11.43 ± 1.61	10.61 ± 1.13	2.613	0.029 *
	ACS-oVEMP	10.45 ± 0.81	11.12 ± 1.11	−2.88	0.018 *
	GVS-oVEMP	8.34 ± 0.84	8.19 ± 0.73	0.813	0.479
N23/P1 (ms)	ACS-cVEMP	23.33 ± 7.69	19.81 ± 1.58	2.954	0.018 *
	GVS-cVEMP	17.65 ± 1.57	16.94 ± 1.49	1.987	0.099
	ACS-oVEMP	15.63 ± 1.14	15.30 ± 1.34	1.107	0.396
	GVS-oVEMP	11.74 ± 0.99	11.31 ± 0.99	1.769	0.131
Interval (ms)	ACS-cVEMP	7.66 ± 7.60	6.43 ± 1.00	1.055	0.396
	GVS-cVEMP	6.22 ± 1.18	6.33 ± 1.01	−0.446	0.657
	ACS-oVEMP	5.18 ± 1.09	4.18 ± 0.76	4.674	<0.001 *
	GVS-oVEMP	3.39 ± 0.64	3.12 ± 0.52	1.948	0.099

**Table 4:** Comparison of VEMP amplitudes between the ADHD and TD groups. Asterisks (*) indicate *p* < 0.05.

Parameter	VEMP	ADHD Group (n = 45)	TD Group (n = 34)	T-Value	*p*-Value (FDR)	Cohen’s d
Amplitude (μV)	ACS-cVEMP	348.16 ± 181.27	112.74 ± 73.65	7.746	<0.001 *	1.63
	GVS-cVEMP	151.45 ± 91.91	135.30 ± 59.89	0.927	0.439	0.2
	ACS-oVEMP	5.05 ± 4.02	4.50 ± 3.16	0.657	0.548	0.15
	GVS-oVEMP	6.04 ± 2.69	4.69 ± 2.09	2.372	0.047 *	0.55

## Data Availability

The original contributions presented in this study are included in the article. Further inquiries can be directed to the corresponding authors.
